# Evaluation of Cryopreserved Primary Swine Macrophages as a Substrate for African Swine Fever Virus Replication

**DOI:** 10.3390/mps9030067

**Published:** 2026-04-24

**Authors:** Vivian K. O’Donnell, Andrew Schoepke, Heather Petrowski, Leslie Blakemore, Douglas P. Gladue, Bonto Faburay, Manuel V. Borca

**Affiliations:** 1 Foreign Animal Disease Diagnostic Laboratory (FADDL), National Veterinary Services Laboratories, Animal and Plant Health Inspection Service, U.S. Department of Agriculture, Plum Island Animal Disease Center, Orient, NY 11957, USA; andrew.schoepke@usda.gov (A.S.); heather.m.petrowski@usda.gov (H.P.); leslie.blakemore@usda.gov (L.B.); bonto.faburay@usda.gov (B.F.); 2 National Bio- and Agro-Defense Facility (NBAF), National Veterinary Services Laboratories, Animal and Plant Health Inspection Service, U.S. Department of Agriculture, Manhattan, KS 66506, USA; 3 Agricultural Research Service, U.S. Department of Agriculture, Plum Island Animal Disease Center, Orient, NY 11957, USA; doug@seeklabs.com (D.P.G.); manuelvborca@hotmail.com (M.V.B.)

**Keywords:** African swine fever, macrophages, cryopreservation, virus isolation, diagnosis

## Abstract

African swine fever (ASF) is a highly contagious and often fatal viral disease of pigs that poses serious economic consequences to the swine industry due to its high mortality rate and rapid spread. Currently, the identification of infectious ASF virus (ASFV) is the confirmatory test when clinical samples are positive for ASFV by any other diagnostic methods. Detection of infectious ASFV requires the availability of primary swine macrophage cultures as a cell substrate. We demonstrate here that cryopreserved swine primary macrophages are a suitable cell substrate for the detection, isolation and propagation of ASFV, showing similar results as when fresh swine macrophages are utilized. The possibility of using cryopreserved macrophages for detecting infectious ASFV would improve the efficacy of diagnostics in ensuring the availability of macrophage cell cultures during an emergency response.

## 1. Introduction

African swine fever virus (ASFV) is the causative agent of African swine fever (ASF), a highly contagious viral disease of pigs characterized by fever, hemorrhage, ataxia, and severe depression, resulting in serious economic losses due to its high mortality rate [1]. ASFV (*Asfivirus haemorrhagiae*) is the only member of the genus *Asfivirus* within the family *Asfarviridae*, an enveloped virus containing a double-stranded DNA genome of 170–193 kilobase pairs (kbp), encoding more than 150 open reading frames (ORFs) [2,3]. ASF was described for the first time in Kenya, Africa, in 1921 [4]. Following the introduction of ASFV in the Caucasus region in 2007, the virus spread through Russia and several countries in Western Europe. In 2018, ASFV was introduced to China, with the virus spreading rapidly over large distances. In 2021, the occurrence of ASF was reported in the Dominican Republic and Haiti, marking the first detection in the Western hemisphere since 1984. Most recently, ASFV has been detected in Papua New Guinea, South Korea, Spain and Latvia [5,6,7,8,9]. The increased number of countries with the disease poses a significant threat to countries that are free of the disease by possible introductions of ASFV via the legal and illegal importation and trade of contaminated pork products and waste. At present, there is no treatment or vaccine available worldwide for ASF, although regionally available live attenuated vaccines have begun to emerge commercially [10,11,12,13]. At this time, vaccination is strictly prohibited in the European Union and other countries; therefore, disease control is currently dependent on timely and reliable diagnosis, along with well-planned surveillance to control the spread of the disease. Preliminary diagnosis of ASFV is done using real-time PCR to detect the viral genome, followed by virus isolation for the confirmation of an active infection and whole genome sequencing for virus characterization [14,15]. The natural target cells for ASFV are porcine monocytes and macrophages. For virus isolation, primary swine macrophage cultures derived from swine blood have been historically used to stably grow virulent and recombinant ASF viruses [16,17,18]. However, traditional cell culture of macrophages is a process that may not be available for every laboratory, since it is time-consuming and requires specialized facilities, trained personnel, and dedicated animal husbandry resources to maintain pigs for blood donation.

Numerous efforts have been made to develop and establish immortalized cell lines to grow ASFV to study the different biological properties of the virus as well as for the development of diagnostic systems and for the generation of recombinant viruses as vaccine candidates [19]. In most cases, the virus needs several passages to gain the ability to replicate in the cell line, which usually leads the virus to incorporate genetic modifications that can result in a decreased virulence in pigs and in reduced ability of the virus to efficiently replicate in macrophages.

Rai et al. (2021) described MA-104, a kidney epithelial cell line, as a suitable substrate for the detection of ASFV in field samples. ASFV-infected MA-104 cells can form rosettes with similar characteristics to rosettes observed with infected fresh macrophages [20]. However, further studies indicated that MA-104 has a lower sensitivity to infection with ASFV when compared to fresh macrophages and that the virus needs adaptation, usually after a process involving successive passages [21]. Another cell line that seemed promising for ASFV replication was the growth factor-dependent ZMAC-4 porcine macrophage cell line derived from fetal pig lung macrophages. The ZMAC-4 cell line has also been described as a suitable cell line for research on ASFV [22]; however, it has been shown that this cell line presents some difficulties in establishing cell cultures that efficiently and steadily grow; therefore, implementation of this cell line for diagnosis for a rapid response may present some complications. Another cell line that has shown promising results is the immortalized primary porcine kidney macrophage (IPKM) cell line. This cell line has shown high susceptibility with several ASFV isolates, comparable to those observed in porcine alveolar macrophages, without needing an adaptation period; furthermore, it is capable of forming rosettes with a hemadsorption assay (HA) [19,23]. Nevertheless, more studies seem necessary to evaluate and validate these cell lines in their capacity to detect, isolate and propagate ASFV with a susceptibility similar to that of primary swine macrophages without any need for an adaptation process. Additionally, some of these cell lines are not commercially available; therefore, the availability of these cell lines is limited and may be restricted by collaborative agreements.

The diagnostic procedures for ASF, as described in the WOAH Terrestrial Manual, include virological and serological methods [15]. The identification of the virus in clinical samples, principally whole blood, is performed using real-time polymerase chain reaction (rt-PCR), although this technique cannot confirm the presence of live virus. Virus isolation, utilizing primary swine macrophage cultures, is vital for the confirmation of the presence of viable virus. The inoculation of primary swine macrophage cultures derived from blood or bone marrow cultures is the confirmatory test when the presence of ASFV is detected by other methods, which is particularly important for the confirmation of a primary outbreak of the disease in an ASF-free area [15]. Therefore, the ready availability of swine macrophage cell cultures is a critical issue in any laboratory devoted to the diagnosis of ASFV in clinical samples. This requirement highlights the need to evaluate cryopreservation as an alternative method to reduce the time and cost associated with frequent preparation of fresh swine macrophages, by enabling cells to be prepared in large batches and stored for future laboratory use to detect, isolate and propagate ASFV. These results also open the possibility for central laboratories to take charge of the large-scale production of frozen swine macrophage stocks. These frozen stocks could be shipped to diagnostic laboratories that, although unable to produce fresh cultures, can receive and maintain frozen macrophages and thus be ready for processing clinical samples.

In this study, we demonstrate that cryopreserved swine macrophages can provide valuable features for the detection, isolation and propagation of ASFV with an efficacy comparable to fresh swine macrophage cultures. The capability to generate large stocks of cryopreserved macrophages allows for constant and immediate access to cells for diagnosis during an outbreak or an emergency response, including the capability to transfer the cells to veterinary diagnostic laboratories if needed. Cryopreserved macrophages are also a promising alternative for research applications.

## 2. Materials and Methods

### 2.1. Primary Swine Macrophage Cultures Derived from Blood

Primary swine macrophage cultures were prepared from heparin-treated swine blood, obtained from a total of six 250–300 pound commercial breed Yorkshire females (Thomas D. Morris, Reisterstown, MD, USA) 5–6 months old, as described elsewhere [16]. Female swine were randomly chosen for this experiment as blood donors that were euthanized and terminally bled under biosafety level 3 conditions in the animal facilities at Plum Island Animal Disease Center, following a strict protocol approved by the Institutional Animal Care and Use Committee (IACUC Protocol# 205.01-23-R). Briefly, heparin-treated swine blood was incubated in a 37 °C water bath for 1–2 h to allow sedimentation of the erythrocyte fraction. Mononuclear leukocytes were separated by a Ficoll-Paque (Pharmacia, Piscataway, NJ, USA) density gradient (specific gravity, 1.079). The monocyte/macrophage cell fraction was cultured in plastic Primaria tissue culture flasks (Corning, NY, USA) containing macrophage-culturing media composed of RPMI 1640 Medium (Life Technologies, Carlsbad, CA, USA) supplemented with 30% L929 supernatant as a natural source of macrophage colony-stimulating factor to support monocyte differentiation into macrophages [24], 20% heat-inactivated–gamma-irradiated fetal bovine serum (FBS) (HyClone, Logan, UT, USA), 1 X antibiotics/antimycotics (Gibco, ThermoFisher Scientific, Waltham, MA, USA), 1 X gentamicin (Gibco, ThermoFisher Scientific, Waltham, MA, USA), and 30% complementary plasma to maintain a more natural environment during differentiation. Cultures were incubated for 24 h at 37 °C under 5% CO_2_. Adherent cells were detached the next day using 10 mM EDTA in Dulbecco’s phosphate-buffered saline (DPBS) (Gibco, ThermoFisher Scientific, Waltham, MA, USA). After discarding the culture supernatant, cells were first washed once with 10 mL of 10 mM EDTA in DPBS, after which 10 mL of fresh 10 mM EDTA-DPBS was added. Flasks were incubated at 37 °C for 20 min to allow detachment. Detached cells were resuspended in FBS, and washed twice with macrophage wash medium, composed of RPMI 1640 Medium, 1 X antibiotics/antimycotics, and 1 X gentamicin. Cells were re-seeded into Primaria plates for use in assays 24 h later or cryopreserved for further evaluation.

### 2.2. Cryopreservation of Primary Swine Macrophages Derived from Blood

A total of six cryopreserved primary swine macrophage stocks were prepared and evaluated in parallel with fresh swine macrophages. Briefly, primary swine macrophages were cryopreserved in 7% DMSO (Sigma-Ald. rich, Inc., St. Louis, MO, USA) and 93% heat-inactivated-gamma-irradiated FBS. Briefly, after detaching adherent macrophage cells from the Primaria flasks, cell suspensions were washed twice with macrophage wash medium composed of RPMI 1640 Medium, 1 X antibiotics/antimycotics, and 1 X gentamicin by centrifugation at 240× *g* for 10 min at room temperature (RT). After the second wash, supernatants were discarded, and the cell pellets were resuspended in pre-chilled 7% DMSO/93% FBS. Aliquots at 1 × 10^7^ cells/mL were dispensed into cool-down cryovials, placed in a Mr. Frosty™ Freezing container (ThermoFisher Scientific, Waltham, MA, USA), stored at −70 °C for 48 h, and then transferred to liquid nitrogen for long-term storage. For seeding and testing the cryopreserved macrophages, the cryovials were removed from liquid nitrogen and quickly thawed in a water bath at 37 °C. Cells were pipetted out of the vial into a 15 mL conical tube and then washed twice in macrophage complete media composed of RPMI 1640 Medium with 30% L929 supernatant, 20% heat-inactivated–gamma-irradiated FBS, 1 X antibiotics/antimycotics, and 1 X gentamicin by centrifugation at 240× *g* for 10 min. After the second wash, the cell pellet was resuspended in the same macrophage complete media and viability was determined using Trypan blue (ThermoFisher Scientific, Waltham, MA, USA) to determine membrane integrity [25]. Live and dead cells were counted manually using a Neubauer hemacytometer counting chamber (ThermoFisher Scientific, Waltham, MA, USA) following the manufacturer’s instructions. Following viability and cell counting determination, the cells were diluted to the desired concentration and seeded directly into Primaria 96-well plates. The cells were maintained in the same macrophage complete media for use in assays 24 h after seeding. No alternative culture system was used during this period.

### 2.3. Red Blood Cells for Hemadsorption Test

Red blood cells (RBCs) were obtained from heparin-treated swine blood during the preparation of the primary swine macrophage cultures. Briefly, heparin-treated swine blood was incubated at 37 °C for 1–2 h to allow sedimentation of the erythrocyte fraction. At that time, 35 mL of the erythrocyte fraction were removed, resuspended in 1 X of DPBS and washed twice by centrifugation at 240× *g* for 10 min at RT. After the second wash, the RBCs were resuspended in DPBS at 20% final concentration and stored at 4 °C until further use. RBCs were used no more than 7–14 days after obtained. As an alternative source of swine RBCs, heparin-treated swine blood from different pigs was obtained from the National Center of Animal Health (NCAH), Ames, IA, for comparison with the homologous swine RBCs on the HA test. RBCs obtained from different pigs from which the macrophages were produced were washed and stored similarly to the homologous swine RBCs and were referred to as heterologous RBCs. Furthermore, commercially available porcine RBCs (Innovative Research, Inc., Novi, MI, USA) were evaluated as an alternative source of swine RBCs for when a source of swine RBC from homologous or heterologous pigs might not be available.

### 2.4. Viruses

ASFV isolates Georgia 2007/1, Lisbon-60, Killean-III, Kimakia-64, Dominican Republic-2 (DR-2), Dominican Republic-21 (DR-21) and Haiti-21 were provided by the U.S. Department of Agriculture, National Veterinary Services Laboratories’ Foreign Animal Disease Diagnostic Laboratory (USDA-NVSL-FADDL), Plum Island Animal Disease Center (PIADC), New York. ASFV isolates Malawi Lil-20/1 and Pretoria-4/1 were provided by the U.S. Department of Agriculture, Agriculture Research Service at PIADC. The complete historical passage information was not available for DR-2, Killean-III and Kimakia-64 isolates, since these were archive samples from the institutional repository. However, it is understood that all the isolates were propagated only in primary swine macrophages. For all other isolates (Georgia 2007/1, Lisbon-60, DR-21, Haiti-21, Malawi Lil-20/1 and Pretoria-4/1), the original material was used to recover virus, and propagation was performed exclusively in primary swine macrophages with no more than two passages in these cells. For the experiments described in the manuscript, each isolate was passed once in primary macrophage cultures derived from blood to generate viral stocks that were stored at −70 °C until used.

### 2.5. Porcine Blood Samples from ASFV Experimentally Infected Pigs

EDTA-treated blood samples were collected from a total of twenty-four pigs. Twelve of them were experimentally infected with ASFV Georgia 2007/1 and the other twelve were experimentally infected with ASFV Lisbon-60. All samples were taken between 6 and 7 days post-inoculation (d.p.inoc.), when clinical signs of disease were observed. Samples were provided by the Foreign Animal Disease Diagnosticians (FADD) School, USDA-NVSL-FADDL, PIADC. Additionally, ten EDTA-treated blood samples from a naïve domestic pig population were provided by the Diagnostic Service Section (DSS), FADDL as negative samples.

### 2.6. Virus Titration

Virus titrations were performed on fresh and cryopreserved primary swine macrophage cultures side by side, using cells 24 h after seeding, to determine the susceptibility of infection for comparison. Briefly, ASFV isolates and EDTA-treated blood samples were 10-fold serial diluted with macrophage complete media. A volume of 50 μL of each dilution was added per well to primary swine blood macrophages cultured in Primaria 96-well tissue culture plates at a density of 5 × 10^6^ cells/plate. Detection of ASFV infected cells was determined by HA. Rosettes are formed in the presence of RBCs, which were added 24 h after the inoculation at a volume of 10 μL per well. Final readings were performed between 7 and 10 days post infection (d.p.i.), and virus titers were calculated by the Reed and Muench method and expressed as HAD_50_/mL, with the limit of detection corresponding to the lowest dilution in which HA could be detected [26].

### 2.7. Growth Curve

Fresh and 1-month cryopreserved macrophages were cultured in 6-well Primaria tissue culture plates at a concentration of 1 × 10^6^ cells/well and incubated for 24 h. Macrophage cultures were inoculated in triplicate with ASFV Georgia 2007/1 or Lisbon-60 at a multiplicity of infection (MOI) of 0.01. After 1 h incubation at 37 °C, the inoculum was removed, and the cells were washed two times with macrophage complete medium. Macrophage culture medium (2 mL) was then added to each well and the plates were incubated at 37 °C in a 5% CO_2_ incubator. Supernatants were collected at 0 h post-inoculation (h.p.inoc.) and 1, 2, 5, 7 and 9 d.p.inoc. and stored at −70 °C until further evaluation. Viral titers were determined in fresh macrophage cultures and expressed as HAD_50_/mL as described above.

### 2.8. Repeatability Studies

Fresh isolated macrophages and two separate stocks of cryopreserved macrophages, 2 months and 6 months post-cryopreservation, were used to seed 96-well Primaria tissue culture plates at a density of 5 × 10^6^ cells/plate. Ten-fold serial dilutions of ASFV Georgia 2007/1 were prepared in triplicate and used to inoculate the plates seeded with fresh or cryopreserved macrophages. The experiment was technically reproduced over three consecutive days to determine repeatability and reproducibility of the performance of cryopreserved macrophages as compared with fresh macrophages for ASFV replication.

## 3. Results and Discussion

### 3.1. Viability of Cryopreserved Primary Swine Macrophages

**Figure 1:**
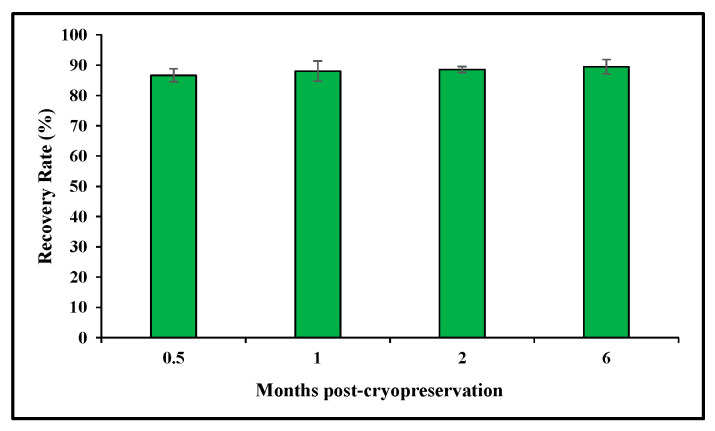
Viability of cryopreserved swine macrophages. Two different stocks of cryopreserved swine macrophages from each time point were quickly thawed, and viability was determined by Trypan blue staining. The results indicate the mean and standard deviation calculated across all measurements from the two stocks at each time point. The recovery rate was expressed as a percentage of live cells over the total number of cells.

Two different stocks of cryopreserved primary swine macrophages, which had been frozen for 0.5 month, 1 month, 2 months or 6 months, were used for evaluating cell viability. In this experiment, two vials from each stock at each time point were thawed and cell viability was determined using Trypan blue. The results showed a survival yield ranging between 80% and 90%, which agrees with expected survival rates for cryopreserved cells, and remained stable regardless of the length of the cryopreservation [25,27] (Figure 1).

### 3.2. Susceptibility of Cryopreserved Macrophages to Infection with ASFV and Evaluation by HA Using Homologous or Heterologous Swine Red Blood Cells

**Figure 2:**
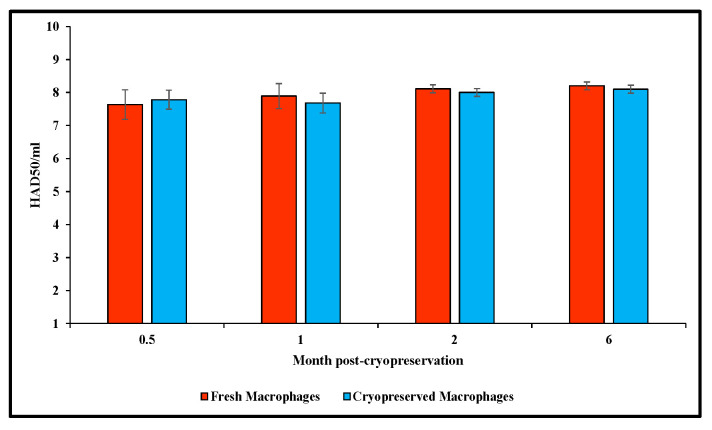
Susceptibility of cryopreserved macrophage cultures to infection with ASFV Georgia 2007/1 as compared with fresh macrophage cultures. Two different stocks of cryopreserved swine macrophages at each time point were used. The results indicate the mean and standard deviation calculated across all measurements from the two cultures at different times. Titers are expressed in log_10_ HAD_50_/mL.

Next, we evaluated the susceptibility of the cryopreserved primary swine macrophage cultures to infection with ASFV Georgia 2007/1 compared with fresh primary swine macrophages. For these experiments, the fresh and the stocks of cryopreserved macrophage cells that had been tested previously were used. When 0.5-, 1-, 2- and 6-month post-cryopreservation cultures were compared with fresh macrophage cultures, ASF Georgia 2007/1 reached comparable titers across all conditions. Observed differences fell within the expected variability of the HA. Therefore, we can conclude that ASFV Georgia 2007/1 reached similar titers in cryopreserved macrophage cultures as those compared with fresh macrophage cultures, independent of the time post-freezing and the pig used for the preparation of the cryopreserved macrophages (Figure 2).

**Figure 3:**
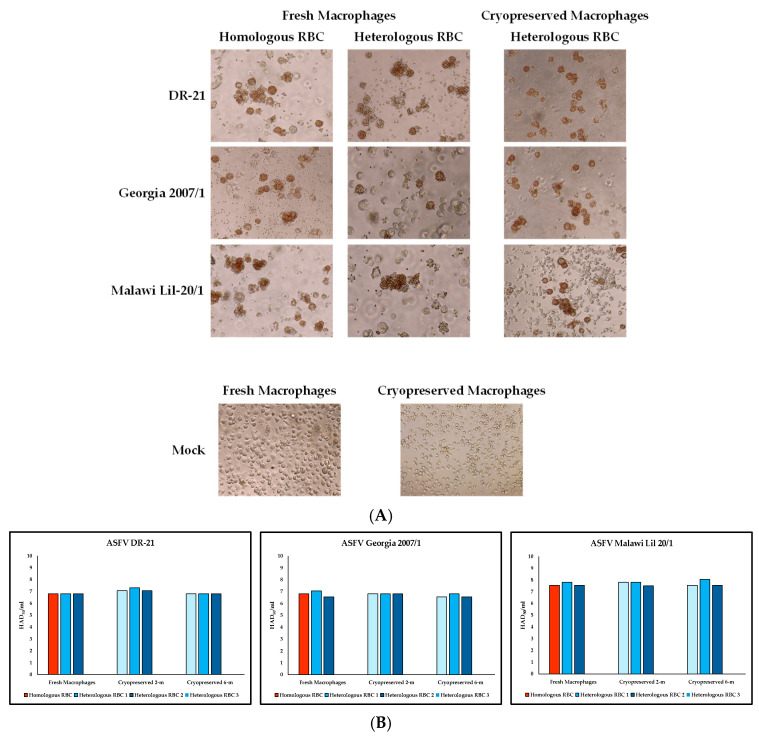
Hemadsorption assay (HA) in fresh and cryopreserved primary swine macrophages. (**A**) Cell cultures were mock-infected or infected with ASFV Georgia 2007/1. At 24 h.p.i., homologous or heterologous red blood cells were added into the cultures, and the detection of virus infection was determined by HA by the presence of rosettes. Differences in apparent cell or rosette size are due to the biological variation and image cropping, not to differences in magnification. Images captured by Evo microscope, 20× magnification. (**B**) Fresh macrophages and two stocks of macrophages cryopreserved for 2 and 6 months were infected with three different ASFV isolates: DR-21 (**left panel**), Georgia 2007/1 (**middle panel**), and Malawi Lil 20/1 (**right panel**). Cryopreserved macrophages were tested using different sources of RBCs. Titers were expressed as log_10_ HAD_50_/mL as determined by the formation of rosettes in the presence of homologous or heterologous RBCs.

Most of the ASFV field isolates cause HA of swine RBCs to infected macrophages, forming rosettes [16]. Therefore, the HA is a widely used simple and accurate method to identify ASFV-infected macrophages. We then evaluated if ASFV-infected cryopreserved macrophages are still able to form rosettes as efficiently as freshly produced macrophages. Using RBCs obtained from a different pig than the macrophage origin could be an issue in the use of cryopreserved macrophages and might bring some unforeseen technical problems. To analyze this issue, RBCs from homologous and heterologous sources to the macrophages were tested in cultures infected with three different ASFV isolates: ASFV Georgia 2007/1, DR-21—both genotype II (GII)—and Malawi Lil 20/1, GVIII. First, homologous and heterologous RBCs were tested using fresh macrophages infected with the three different ASFV isolates. ASFV-infected fresh macrophages formed rosettes with similar morphology when either homologous or heterologous RBCs were used. Additionally, the final titers for all three ASFV isolates used in the experiment were consistent independent of the source of RBCs used, indicating that homologous or heterologous RBCs can be used for the detection of ASFV-infected cells by HA (Figure 3A,B). Two different stocks of cryopreserved macrophages, two and six months post-cryopreservation, infected with the same three ASFV isolates were evaluated for rosette formation only with heterologous RBCs, since homologous RBCs were not available at the time when the cells were thawed. Results showed that all ASFV-infected cryopreserved cultures were able to form rosettes with a similar rosette phenotype as that which formed in ASFV-infected fresh macrophages. Again, no significant differences in the final titers were observed for all three ASFV isolates, regardless of the RBC source utilized, source of macrophages or months post-cryopreservation, which was consistent with the observations seen with fresh macrophages (Figure 3A,B). Furthermore, commercially available porcine RBCs were tested in ASFV Georgia 2007/1-infected macrophages as an alternative source of RBCs when no source of fresh RBCs from pigs is available—in this situation, comparable rosette morphology and similar final titers, log_10_ 6.4 HAD_50_/mL and 6.44 HAD_50_/mL, for fresh and cryopreserved macrophages, respectively, were observed. Although only HAD-positive isolates were included in this study, a clear cytopathic effect was observed in infected macrophage cultures, supporting the potential applicability of this approach to HAD-negative isolates. Mock-infected cultures, using macrophage complete media as inoculum, were negative with no rosettes detected (Figure 3A).

Overall, our data indicated that cryopreserved primary swine macrophages are susceptible to infection with ASFV and maintain the capacity to form HA rosettes similarly to ASFV-infected fresh macrophage cultures, regardless of whether homologous, heterologous, or commercial porcine RBCs are used.

### 3.3. Viral Growth in Cryopreserved Macrophage Cells

**Figure 4:**
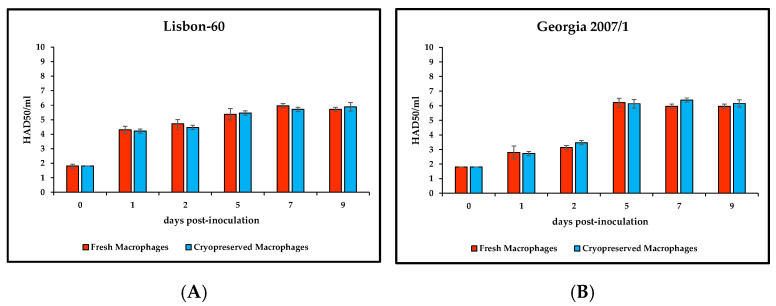
Comparison of ASFV Lisbon-60 and Georgia 2007/1 growth kinetics in fresh and cryopreserved macrophages. Cell cultures were infected with ASF Lisbon-60 (**A**) or Georgia 2007/1 (**B**) at an MOI of 0.01. Cell culture supernatants were collected at different times post-infection and viral yields determined as described in Materials and Methods. Virus titers are expressed in log_10_ HAD_50_/mL. Each time point represents three biological replicates and the results indicate the means and standard deviations of these experiments.

We further evaluated the ability of two ASFV isolates, Lisbon-60 and Georgia 2007/1, as representatives of genotypes I and II, respectively, to replicate in 3-month cryopreserved macrophages as compared to fresh macrophages. Macrophage cultures were infected at a MOI of 0.01 and virus yields were evaluated at 0, 1, 2, 5, 7 and 9 days post-infection. Results demonstrated that ASFV Lisbon-60 replicates similarly in fresh and 3-month cryopreserved macrophages. Slight differences were observed early after infection (2 d.p.i.), but these fell within the normal variability of the HA test. Toward the end of the experiment, comparable maximal titers of 5.72 HAD_50_/mL and 5.88 HAD_50_/mL were observed for fresh and cryopreserved macrophages, respectively. Likewise, growth kinetics of ASFV Georgia 2007/1 were similar in fresh and cryopreserved macrophages. A slight delay was noted at 1 d.p.i., when compared to fresh macrophages. Nevertheless, the titers stayed consistent at 2 d.p.i. and remained stable toward the end of the experiment, showing a maximal titer of 6.05 HAD_50_/mL and 6.3 HAD_50_/mL for fresh and cryopreserved macrophages, respectively, at 9 d.p.i. (Figure 4). Taken together, our data indicated that both viruses replicate similarly in fresh and cryopreserved primary swine macrophage cultures.

### 3.4. Repeatability Performance of Cryopreserved Macrophages to Grow ASFV

**Figure 5:**
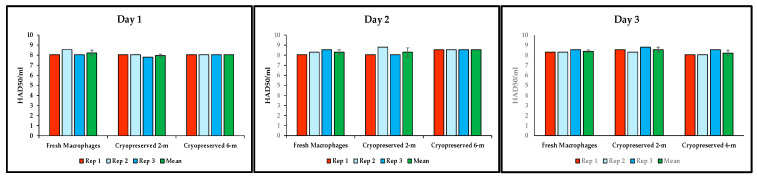
Repeatability performance of cryopreserved macrophages compared to fresh macrophages. Fresh and 2 months and 6 months cryopreserved macrophages were infected with 10-fold dilutions of ASFV Georgia 2007/1 in triplicate for 3 consecutive days (**left**, **middle** and **right panels**). The presence of virus-infected cells was determined by HA and titer values expressed as log_10_ HAD_50_/mL. The results indicate the three individual titers per replicate (Rep) per day, and the means and standard deviations for the three experiments. m: months.

Furthermore, we comparatively evaluated the repeatability performance of the cryopreserved macrophages and fresh macrophages to detect ASFV Georgia 2007/1, the isolate responsible for the current outbreaks in Europe and Asia. Our results indicated that the two different stocks of cryopreserved macrophages (2 and 6 months post cryopreservation) performed similarly all three days, with comparable final titers to those obtained with fresh macrophages (Figure 5). In addition, the viral titers were very consistent between replicates, with only one replicate showing 0.5 log_10_ difference when the fresh macrophages and the 2-month cryopreserved cultures were compared at day 3 (Figure 5 right panel).

### 3.5. Susceptibility of Cryopreserved Primary Swine Macrophages to Detect Infectious ASFV in Blood Samples Compared to That of Fresh Primary Swine Macrophages

The sensitivity of the cryopreserved primary swine macrophages was assessed in comparison to that of fresh primary swine macrophages to detect infectious ASFV in blood samples, a sample type that would typically be collected from a suspected case of ASFV. For this experiment, a total of 24 whole-blood EDTA samples from different pigs experimentally infected with ASFV, taken between 6 and 7 d.p.inoc., were processed. Out of the twenty-four samples, twelve corresponded to pigs infected with ASFV Lisbon-60 (samples 1 to 12) and twelve to pigs infected with ASFV Georgia 2007/1 (samples 13 to 24), as representative viruses of ASFV genotypes I and II, respectively.

**Figure 6:**
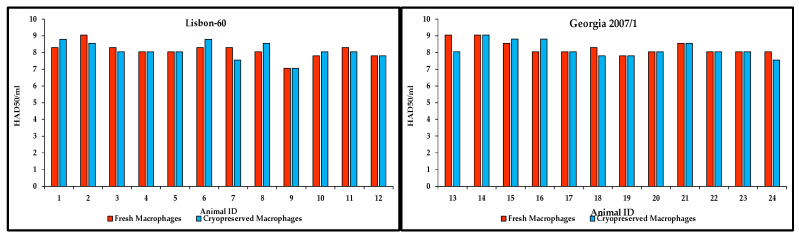
Comparison of the titration of whole blood EDTA clinical samples in fresh and cryopreserved primary swine macrophages. EDTA-blood samples from pigs experimentally infected with either ASFV Lisbon-60 (Animal ID 1 to 12, **left panel**) or Georgia 2007/1 (Animal ID 13 to 24, **right panel**) were evaluated for the detection of infectious ASFV by virus titration performed in parallel in fresh and cryopreserved primary swine macrophages. Titers are expressed in log_10_ HAD_50_/mL.

All pigs showed clinical signs of the disease when the samples were collected, similarly to samples that would likely be taken during a suspected outbreak of the disease. Samples were titrated side by side in cell cultures of fresh and cryopreserved macrophages (2.5 months of cryopreservation). Titer values for the 12 blood samples from the animals infected with Lisbon-60 were similar when both cultures, fresh and cryopreserved, were compared (Figure 6). Four of these samples showed titer values in cryopreserved macrophages, with 0.5 or less log_10_ difference as compared with those obtained with fresh macrophages. Out of the eight remaining samples, four showed the same titers in both cell cultures, while the remaining last four samples showed higher titers when cryopreserved macrophages were used (again, with differences of 0.5 log_10_ or less). In the evaluation of the blood samples collected from pigs experimentally infected with ASFV Georgia 2007/1, seven out of the twelve samples tested showed the same titers in both macrophage cultures. Two samples showed higher titers (0.5 or less log_10_) in cryopreserved macrophages than in the fresh ones, while the remaining three samples showed higher titers in the fresh macrophage cultures than in the cryopreserved cells; one showed a difference of 1-log_10_; and two samples a difference of less than 0.5 log_10_ (Figure 6). Additionally, ten EDTA-blood samples, collected from naïve domestic swine populations, were included as negative control to assure no false positive results were observed when cryopreserved macrophages were used. All ten samples tested negative in both fresh and cryopreserved macrophage cultures showing no rosette formations. Therefore, the use of cryopreserved macrophages for detecting the presence of infectious ASFV particles produces similar results to those obtained by using freshly isolated macrophage cell cultures with no false positives detected.

Furthermore, we evaluated another set of 21 EDTA-blood samples from pigs experimentally infected with the same two ASFV isolates. At this time, the 21 blood samples were evaluated following the protocol for ASF-Virus isolation described by FADDL (PIADC), which directs blood samples to be diluted 1/10–1/100 with at least four replicates per dilution, following WOAH recommendations [15], and the results were expressed as positive or negative as indicated by the presence or absence of rosettes. All 21 EDTA-blood samples tested positive at both dilutions (1/10 and 1/100) for all four replicates, with a complete correlation between fresh and cryopreserved macrophages (Table 1). Taking together these two sets of experiments, we concluded that cryopreserved macrophages maintain the capability of fresh macrophages to detect infectious ASF virus by HA, making the cryopreserved cells a suitable alternative to be used for the detection of infectious ASFV in whole-blood EDTA clinical samples when performing the ASFV isolation protocol.

### 3.6. Susceptibility of Cryopreserved Primary Swine Macrophages to Different ASFV Isolates as Compared to Fresh Primary Swine Macrophages

**Figure 7:**
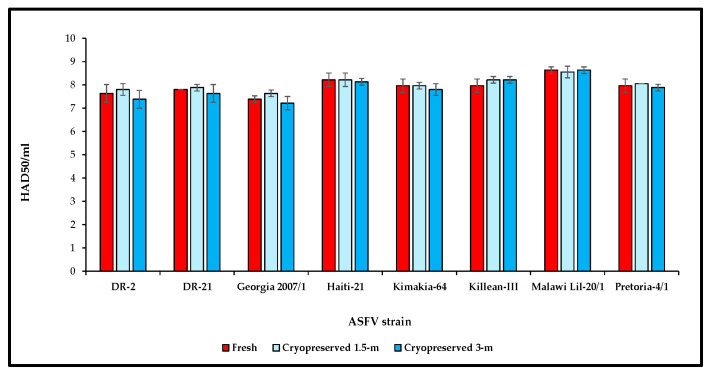
Comparison of virus titration of different ASFV isolates in fresh and cryopreserved primary swine macrophages. Titers were determined by presence of rosettes and expressed as log_10_ HAD_50_/mL. Each virus isolate was tested using three biological replicates and standard deviation of the three replicates were calculated. m: months.

Susceptibility of cryopreserved primary swine macrophages to genetically distinct ASFV isolates DR-2 (GI), DR-21 (GII), Georgia 2007/1 (GII), Haiti-21 (GII), Killean-III (GX), Kimakia-64 (GI), Malawi Lil-20/1 (GVIII), and Pretoria-4/1 (GXX), was comparatively evaluated to that of fresh primary swine macrophages. Two different primary swine cryopreserved macrophage stocks with 1.5 and 3 months of cryopreservation were infected with each isolate in triplicate, and final titers were expressed as log_10_ HAD_50_/mL. A similar set of plates seeded with freshly isolated macrophages was used as a control. Our results demonstrated that cryopreserved primary swine macrophages are susceptible to infection with all eight ASFV isolates tested and maintain comparable sensitivity for detection of infectious particles at similar levels to that of the fresh macrophages. All the ASFV isolates reached similar titers, regardless of the time post-cryopreservation or the source pig for the macrophage preparation (Figure 7).

### 3.7. Evaluation of Long-Term Cryopreserved Macrophages for the Detection of Infectious ASFV

To ensure the dependable availability of cryopreserved macrophages that can efficiently detect the presence of infectious ASFV, we evaluated stocks of macrophages that had been cryopreserved for different lengths of time. Cryopreserved macrophage stocks were evaluated at 18, 68, and 72 months after cryopreservation and compared to fresh and 2-month old cryopreserved stocks. The viability of all the cryopreserved stocks tested was evaluated by Trypan blue staining after thawing the cells and before plating; a recovery rate between 82 and 93% was noted for all stocks.

**Figure 8:**
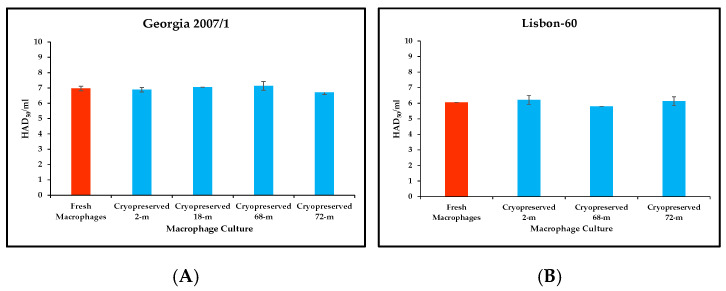
Comparison of the titration of ASFV Georgia 2007/1 (**A**) and Lisbon-60 (**B**) in fresh and cryopreserved macrophages after different cryopreservation times. Titrations were expressed in log_10_ HAD_50_/mL. Each titration was performed using three replicates and the results indicate the means and standard deviations of these replicates.

Ten-fold dilutions of ASF Georgia 2007/1 were prepared in triplicate, and titrations were performed in fresh and 2-, 18-, 68-, and 72- month cryopreserved macrophages, with very similar results in all cultures tested (Figure 8A). Differences in the final titers of ASFV Georgia 2007/1 ranged from −0.3 log_10_ to +0.1 log_10_ for the cryopreserved macrophages when compared with titers obtained using fresh macrophage cultures (Figure 8A). Comparable results were obtained when ten-fold titrations of ASF Lisbon-60, done in triplicate, were evaluated in 2-, 68-, and 72-month cryopreserved macrophages versus fresh macrophages (Figure 8B).

Furthermore, a set of 18 EDTA-blood samples from pigs experimentally infected with either Georgia 2007/1 or Lisbon-60 were tested using the protocol for ASF-Virus isolation described by FADDL. For this experiment, 2- and 72-month post-cryopreservation macrophage stocks were compared along with fresh cultures. Our results showed complete correlation between the virus titers obtained with the two cryopreserved macrophage stocks as compared with fresh macrophages, with all samples being positive in all cultures (Table 2).

Finally, we evaluated the capacity of the different stocks of cryopreserved macrophages frozen at different times (from 1 month to 72 months) to support replication with 1/10 and 1/100 dilutions of eight genetically distinct ASFV isolates. Results demonstrated that all the ASFV isolates evaluated infect cryopreserved macrophages regardless of length of cryopreservation (from 1 to 72 months before use), as all samples showed the formation of RBC rosettes indicating the presence of virus-infected cells. Once again, the results are in complete agreement with the data observed when fresh macrophage cultures were tested (Table 3).

Preliminary diagnosis of ASFV is usually achieved using real-time PCR followed by virus isolation for confirmation of an active infection and sometimes supported by whole genome sequencing for genetic characterization [15]. Porcine monocytes and macrophages, including those derived from swine peripheral blood, alveolae, and bone marrow, are the main target cells for ASFV during infection in the natural swine host [16,17]. Primary swine macrophage cultures have been historically used for virus isolation in diagnostic procedures [15].

Due to the methodological complexity of the procedures used in the preparation of these cell cultures, readily available fresh macrophage cultures may not be a viable option in many diagnostic laboratories; so, the availability of cryopreserved macrophages for diagnostic procedures constitutes a subject of remarkable importance. In this report, we provide evidence that cryopreserved macrophages are able to perform similarly to freshly produced primary macrophage cultures when tested for different aspects of their efficacy in supporting ASFV replication.

As reported here, cryopreserved macrophages show a recovery rate, in terms of viability, of at least 80% (and up to 93%), even after 72 months of cryopreservation, ensuring their robustness for relatively long periods of time. Macrophages cryopreserved for up to 72 months showed similar susceptibility to infection with ASF Georgia 2007/1 and Lisbon-60 isolates as representatives of genotypes I and II, when compared with fresh macrophages. The experimental reproducibility of these results supports the fact that cryopreserved macrophages are a sound alternative to the use of fresh macrophages for detecting infectious ASFV in clinical samples.

The WOAH protocol for ASF virus isolation from field clinical samples recommends inoculating those samples diluted 1/10 in culture media. Results presented in Table 2 and Table 3 demonstrated that all samples tested in this manuscript can be defined as “Positive”, including ASF viral isolates and blood samples from ASFV-infected pigs. None of the clinical samples or virus stocks known to be positive gave false negative results when analyzed using cryopreserved swine macrophage cultures.

Therefore, cryopreserved macrophages maintain susceptibility to infection with ASFV for up to 72 months with a high recovery rate, opening the possibility to generate large stocks of cells and cryopreserve them for long periods of time.

Although alternative cell substrates have been reported, none of them have been recommended by WOAH. MA-104 cells show a consistent 1 log decrease in sensitivity when compared to fresh macrophage cultures, while other alternative substrates such as ZMAC cells are very difficult to grow—particularly after defrosting, indicating that they are problematic for use to support emergency diagnostic procedures. Therefore, at this time, cryopreserved macrophages appear to be the best alternative to the use of fresh macrophages. The IPKM, immortalized primary porcine kidney macrophage, cell line showed promising results in terms of high susceptibility to ASFV replication, similar to that observed in porcine alveolar macrophages, without requiring a process of virus adaptation [19,23]. No evaluation was reported regarding the ability of IPKM cells to support virus growth in comparison with that of swine macrophages derived from peripheral blood. Therefore, more studies are necessary to evaluate and validate these IPKM cell lines as an alternative to the use of primary cultures of swine macrophages.

In summary, results presented here provide a proof-of-concept, based on a limited number of samples, that cryopreserved swine macrophages derived from whole blood can be easily obtained, cryopreserved for several years, and maintained in the laboratory in an efficacious state for the detection, isolation, propagation, and titration of ASFV. Ongoing validation efforts are underway with a large and representative number of samples, including additional ASFV isolates and field samples to further confirm our results across different diagnostic settings.

## Figures and Tables

**Table 1:** Evaluation of EDTA blood samples from swine experimentally infected with ASFV Lisbon-60 or Georgia 2007/1 isolates by ASF-virus isolation at 1/10 and 1/100 dilutions, 4 replicates per dilution, using fresh and 2.5-month cryopreserved macrophages. Results were expressed as Positive or Negative based on the presence of HA.

		Macrophage Culture Used	
Animal ID	ASFV Strain	Fresh	Cryopreserved
1	Lisbon-60	Positive	Positive
2		Positive	Positive
3		Positive	Positive
4		Positive	Positive
5		Positive	Positive
6		Positive	Positive
7		Positive	Positive
8		Positive	Positive
9		Positive	Positive
10		Positive	Positive
11		Positive	Positive
12		Positive	Positive
13	Georgia 2007/1	Positive	Positive
14		Positive	Positive
15		Positive	Positive
16		Positive	Positive
17		Positive	Positive
18		Positive	Positive
19		Positive	Positive
20		Positive	Positive
21		Positive	Positive

**Table 2:** ASF-virus isolation evaluation of EDTA-blood samples from swine experimentally infected with ASFV Lisbon-60 or Georgia 2007/1 using fresh and 2- and 72-month cryopreserved macrophages. Blood samples were inoculated at 1/10 and 1/100 dilutions, 4 replications per dilution. Results were expressed based on the presence of RBC HA.

		Macrophage Culture Used		
		Fresh	Cryopreserved	
Animal ID	ASFV Strain		2-Months	72-Months
1	Lisbon-60	Positive	Positive	Positive
2		Positive	Positive	Positive
3		Positive	Positive	Positive
4		Positive	Positive	Positive
5		Positive	Positive	Positive
6		Positive	Positive	Positive
7		Positive	Positive	Positive
8		Positive	Positive	Positive
9		Positive	Positive	Positive
10	Georgia 2007-1	Positive	Positive	Positive
11		Positive	Positive	Positive
12		Positive	Positive	Positive
13		Positive	Positive	Positive
14		Positive	Positive	Positive
15		Positive	Positive	Positive
16		Positive	Positive	Positive
17		Positive	Positive	Positive
18		Positive	Positive	Positive

**Table 3:** Detection of ASFV isolates using various stocks of cryopreserved macrophages frozen at different times (from 1 month to 72 months). Pos.: Positive, ND: not determined.

ASFV		Fresh	Cryopreserved								
Strain	Genotype		1-m	1.5-m	2-m	3-m	6-m	9-m	18-m	60-m	72-m
DR-2	I	Pos.	Pos.	Pos.	Pos.	Pos.	Pos.	Pos.	Pos.	ND	Pos.
DR-21	II	Pos.	Pos.	Pos.	Pos.	Pos.	Pos.	Pos.	ND	ND	Pos.
Georgia 2007/1	II	Pos.	Pos.	Pos.	Pos.	Pos.	Pos.	Pos.	Pos.	Pos.	Pos.
Killean-III	X	Pos.	Pos.	Pos.	Pos.	Pos.	Pos.	Pos.	Pos.	ND	Pos.
Kimakia-64	I	Pos.	Pos.	Pos.	Pos.	Pos.	Pos.	Pos.	Pos.	ND	Pos.
Lisbon-60	I	Pos.	Pos.	Pos.	Pos.	Pos.	Pos.	Pos.	ND	Pos.	Pos.
Malawi Lil-20/1	VIII	Pos.	Pos.	Pos.	Pos.	Pos.	Pos.	Pos.	Pos.	ND	Pos.
Pretoria-4/1	XX	Pos.	Pos.	Pos.	Pos.	Pos.	Pos.	Pos.	Pos.	ND	Pos.

## Data Availability

All data generated or analyzed during this study are included in the published article. No additional data sets were generated or deposited in external repositories.

## References

[1] Galindo I., Alonso C. (2017). African Swine Fever Virus: A Review. Viruses.

[2] Blome S., Franzke K., Beer M. (2020). African swine fever—A review of current knowledge. Virus Res..

[3] Todd A.K., Hall R.J., Wang J., Peacey M., McTavish S., Rand C.J., Stanton J.-A., Taylor S. (2013). Detection and whole genome sequence analysis of an enterovirus 68 cluster. Virol. J..

[4] Montgomery R.E. (1921). On a form of swine fever occuring in British East Africa (Kenya Colony). J. Comp. Pathol. Ther..

[5] Gonzales W., Moreno C., Duran U., Henao N., Bencosme M., Lora P., Reyes R., Nunez R., De Gracia A., Perez A.M. (2021). African swine fever in the Dominican Republic. Transbound. Emerg. Dis..

[6] Spinard E., O’Donnell V., Vuono E., Rai A., Davis C., Ramirez-Medina E., Espinoza N., Valladares A., Borca M.V., Gladue D.P. (2023). Full genome sequence for the African swine fever virus outbreak in the Dominican Republic in 1980. Sci. Rep..

[7] OIE (2021). African Swine Fever (ASF)—Situation Report.

[8] World Organisation for Animal Health (WOAH) (2025). African Swine Fever in Wild Boars in Spain—Technical Webinar. https://rr-europe.woah.org/en/Events/african-swine-fever-in-spain-epidemiological-situation-laboratory-findings-and-coordinated-response/.

[9] Schambow R.A., Hussain S., Antognoli M.C., Kreindel S., Reyes R., Perez A.M. (2023). Epidemiological Assessment of African Swine Fever Spread in the Dominican Republic. Pathogens.

[10] O’Donnell V., Holinka L.G., Sanford B., Krug P.W., Carlson J., Pacheco J.M., Reese B., Risatti G.R., Gladue D.P., Borca M.V. (2016). African swine fever virus Georgia isolate harboring deletions of 9GL and MGF360/505 genes is highly attenuated in swine but does not confer protection against parental virus challenge. Virus Res..

[11] Borca M.V., Ramirez-Medina E., Silva E., Vuono E., Rai A., Pruitt S., Holinka L.G., Velazquez-Salinas L., Zhu J., Gladue D.P. (2020). Development of a Highly Effective African Swine Fever Virus Vaccine by Deletion of the I177L Gene Results in Sterile Immunity against the Current Epidemic Eurasia Strain. J. Virol..

[12] Tran X.H., Phuong L.T.T., Huy N.Q., Thuy D.T., Nguyen V.D., Quang P.H., Ngôn Q.V., Rai A., Gay C.G., Gladue D.P. (2022). Evaluation of the Safety Profile of the ASFV Vaccine Candidate ASFV-G-ΔI177L. Viruses.

[13] Fan J., Yu H., Miao F., Ke J., Hu R. (2024). Attenuated African swine fever viruses and the live vaccine candidates: A comprehensive review. Microbiol. Spectr..

[14] World Organisation for Animal Health (WOAH) (2024). Addressing African Swine Fever: Protocols and Guidelines for Laboratory Diagnosis.

[15] World Organisation for Animal Health (WOAH) (2024). African Swine Fever. WOAH Terrestrial Manual.

[16] Zsak L., Lu Z., Kutish G.F., Neilan J.G., Rock D.L. (1996). An African swine fever virus virulence-associated gene NL-S with similarity to the herpes simplex virus ICP34.5 gene. J. Virol..

[17] Solikhah T.I., Rostiani F., Nanra A.F.P., Dewi A., Nurbadri P.H., Agustin Q.A.D., Solikhah G.P. (2025). African swine fever virus: Virology, pathogenesis, clinical impact, and global control strategies. Vet. World.

[18] Goatley L.C., Nash R., Netherton C.L., Netherton C.L. (2022). Primary Macrophage Culture from Porcine Blood and Lungs. African Swine Fever Virus.

[19] Meloni D., Franzoni G., Oggiano A. (2022). Cell Lines for the Development of African Swine Fever Virus Vaccine Candidates: An Update. Vaccines.

[20] Rai A., Pruitt S., Ramirez-Medina E., Vuono E.A., Silva E., Velazquez-Salinas L., Carrillo C., Borca M.V., Gladue D.P. (2021). Detection and Quantification of African Swine Fever Virus in MA-104 Cells. Bio-Protocol.

[21] Kwon H.I., Do D.T., Van Vo H., Lee S.C., Kim M.H., Nguyen D.T.T., Tran T.M., Le Q.T.V., Ngo T.T.N., Nguyen N.M. (2022). Development of optimized protocol for culturing African swine fever virus field isolates in MA104 cells. Can. J. Vet. Res..

[22] Portugal R., Goatley L.C., Husmann R., Zuckermann F.A., Dixon L.K. (2020). A porcine macrophage cell line that supports high levels of replication of OURT88/3, an attenuated strain of African swine fever virus. Emerg. Microbes Infect..

[23] Takenouchi T., Kitani H., Suzuki S., Nakai M., Fuchimoto D.I., Tsukimoto M., Shinkai H., Sato M., Uenishi H. (2017). Immortalization and Characterization of Porcine Macrophages That Had Been Transduced with Lentiviral Vectors Encoding the SV40 Large T Antigen and Porcine Telomerase Reverse Transcriptase. Front. Vet. Sci..

[24] Borca M.V., Berggren K.A., Ramirez-Medina E., Vuono E.A., Gladue D.P. (2018). CRISPR/Cas Gene Editing of a Large DNA Virus: African Swine Fever Virus. Bio-Protocol.

[25] Baust J.M., Campbell L.H., Harbell J.W. (2017). Best practices for cryopreserving, thawing, recovering, and assessing cells. Vitr. Cell. Dev. Biol.—Anim..

[26] Reed L.J., Muench H. (1938). A simple method of estimating fifty percent endpoints. Am. J. Hyg..

[27] Liang X., Hu X., Hu Y., Zeng W., Zeng G., Ren Y., Liu Y., Chen K., Peng H., Ding H. (2019). Recovery and functionality of cryopreserved peripheral blood mononuclear cells using five different xeno-free cryoprotective solutions. Cryobiology.

